# The geometry of evolved community matrix spectra

**DOI:** 10.1038/s41598-022-17379-6

**Published:** 2022-08-29

**Authors:** Silja Borring Låstad, Jan O. Haerter

**Affiliations:** 1 Niels Bohr Institute, University of Copenhagen, Blegdamsvej 17, Copenhagen, 2100 Denmark; 2 Leibniz Centre for Tropical Marine Research, Fahrenheitstrasse 6, Bremen, 28359 Germany; 3 Physics and Earth Sciences, Jacobs University Bremen, Campus Ring 1, 28759 Bremen, Germany

**Keywords:** Evolution, Evolutionary theory, Community ecology, Food webs, Complex networks

## Abstract

Random matrix theory has been applied to food web stability for decades, implying elliptical eigenvalue spectra and that large food webs should be unstable. Here we allow feasible food webs to self-assemble within an evolutionary process, using simple Lotka–Volterra equations and several elementary interaction types. We show that, as complex food webs evolve under ${10^5}$ invasion attempts, the community matrix spectra become bi-modal, rather than falling onto elliptical geometries. Our results raise questions as to the applicability of random matrix theory to the analysis of food web steady states.

## Introduction

Ecosystems are networks of species in a habitat where the population of any species generally depends on the populations of all other species. Such complex inter-relations make the species vulnerable to changes in the environment^1–3^. Recent research indicates that human activity drives species to extinction at a rate corresponding to that of a mass extinction^4,5^. Consequently, it is crucial to gain further understanding of the conditions under which ecosystems are stable or susceptible to collapse.

On Earth, the species in ecosystems sustain over many generations, yet stable ecosystems have been found difficult to construct due to their complexity^6–8^. As shown in seminal work^9^, a dynamical system represented by a random Jacobian is exceedingly unlikely to be stable the more the number of species is increased. Hence, if real ecosystems can be represented as random matrices, they are very unlikely to be stable. The situation can seemingly be improved, when making additional assumptions: stabilisation is achieved by self-regulation^10^, where mechanisms such as cannibalism aid in stabilising due the effective reduction of a species’ growth rate with its population size.

Recent work builds on the notion of randomly composed ecosystems^7,11–13^, describing additional ways in which stability can be obtained. Introducing predator-prey interactions into a random matrix framework results in an elliptical eigenvalue spectrum and thus stabilising the food web^7,8^. Randomly exposing a large set of species to one-another and checking for feasible survivors is another path to achieve a coexisting community^12,13^. Real ecosystems exhibit a range of additional interactions and dynamics^14,15^. Additional links, such as those from omnivory or parasitism, have been suggested to yield greater food web stability^16–21^. Conversely, also the lack of omnivory, that is *trophic coherence*^22^, has been shown to promote coexistence.

However, complexity itself can be seen as the result of a lengthy computational task^23^, hence in principle ruling out the “shortcut” via random matrices as a means of achieving plausible community matrices. Departing from food webs that are randomly composed, our current work more closely relates to generative food web models, where invaders sequentially attempt to enter an existing food web and thus shape coexistence through an evolutionary process^24–27^. We build an algorithm that simulates many successive invasion attempts by introducing species one by one with a randomly chosen set of interaction parameters. After each invasion attempt, species populations are allowed to acquire a new steady state—often requiring extinctions of the invader or any of the resident species.

We then ask in how far the community matrices, that is, the Jacobian of the Lotka–Volterra equations evaluated at the fixed points, yield elliptical eigenvalue spectra as predicted by random matrix theory. Using three different invasion mechanisms we show that evolved community matrix spectra are increasingly bi-modal as species richness grows—with many eigenvalues with small and negative real parts, but several eigenvalues showing large negative real parts. Specifically, we first study treelike food webs, where each species can have only one resource, and which are known to become increasingly robust under repeated invasions^28–30^. Second, we contrast to food webs with loops, which can be trophically coherent or omnivorous. In these more complex cases species richness remains bounded and the concept of absolute fitness breaks down, resulting in a distinct evolutionary dynamics. Yet, also here, greater species richness is associated with bi-modal community matrix spectra. In contrast, the spectra obtained from random matrices are uni-modal with a peak at intermediate real parts. Our results hence raise the question, in how far a random matrix framework is applicable to food webs that resulted from an evolutionary process.

## Results and discussion

### Modelling complex evolved food webs

Our interest here is to develop a conceptual comparison between the eigenvalue spectrum of a complex, evolved food web and a random matrix analog. We therefore focus on the widely-used generalised Lotka–Volterra equations for consumer-resource interactions. For simplicity, we further restrict to a single basic nutrient source, and require that species feeding on the basic nutrient source are never omnivorous^31^, e.g., plants do not consume other plants. The original Lotka–Volterra equations^32,33^ describe spatially and temporally homogeneous, consumer-resource relations. The generalised Lotka–Volterra equations^34–36^ can be used to describe the dynamics of larger, more complex food webs, and encode the dynamics of primary producers as

1 $$\begin{aligned} \frac{\dot{S_i}}{S_i} = k_i \left( 1 - \sum _{j=1}^{n_1} S_j \right) - \alpha _i - \sum _{k=n_1+1}^{n} \eta _{ki} S_k, \end{aligned}$$

where $S_i$, $i\in \{1,\dots ,n_1\}$, denote the population densities of primary producers in units of biomass, normalised to the system carrying capacity and $n_1$ denotes the total number of primary producers, $k_i>0$ denote the growth rates of the corresponding primary producer $S_i$, that is, the maximal reproduction rate at unlimited nutrient availability. We use $k_i=k$ for all primary producers. The negative sum on the species $S_j$ encodes logistic growth by accounting for nutrient depletion by all primary producers. For all other species, $S_k$, $k\in \{n_1+1,\dots ,n\}$ with *n* the total number of species in the food web, the equations read

2 $$\begin{aligned} \frac{\dot{S_k}}{S_k} = \sum _{m=1}^{n} \beta _{km}\eta _{km} S_m - \alpha _k - \sum _{p=n_1+1}^{n} \eta _{pk} S_p. \end{aligned}$$

Here, $S_k$ is again measured in units of normalised biomass. In Eqs. (1) and (2), $\alpha _j>0$ is the decay rate of a species $S_j$, representing death not caused by consumption through other species. $\eta _{ki}\ge 0$ is the link-specific interaction strength between consumer $S_k$ and resource $S_i$. On the RHS of either equation, note the final term representing the diminishing effects experienced by each resource species, which is caused by consumption. This term is mirrored by the first term in Eq. (2), which describes the strengthening effect on the consumer side. The coefficients $\beta _{ki}\le 1$ encode link-specific consumption efficiency—that is, potentially incomplete use of energy removed from a resource species by its consumer. $\beta _{ki}=1$ would describe perfect consumption efficiency whereas in real food webs this value is estimated to lie considerably lower^37^. In our simulations we use $\beta _{ki}=\beta$ for all interactions present.

Equations (1) and (2) describe a simplified food web structure where consumption is modelled by the simple Holling type-I response^38^, where consumer resource fluxes scale proportional to the product of consumer and resource biomass density and there are no saturation effects. Moreover, Eqs. (1) and (2) assume that the food web is rigid in that species are incapable of adapting their consumption behaviour to changes within the food web, such as a decreasing population of resources or competition from an invasive species^39^. Yet, these equations allow for a coherent description of the energy fluxes between species and constitute an established framework for complex consumer-resource relations to evolve.

To evolve food webs we simulate Eqs. (1) and (2) numerically. New species are added successively to an existing food web. We assume that invasion attempts occur on a slow timescale, such that equilibrium can be reached before the subsequent invasion attempt, though occasionally, the food web does not converge to its equilibrium state. After each invasion attempt the steady state species vector $\mathbf {S^*}$ is computed. In case of feasibility the eigenvalues of the community matrix are evaluated in order to determine the linear stability of the steady states. If feasibility is not obtained, that is, if $\mathbf {S^*}$ contain negative populations, Eqs. (1) and (2) are integrated numerically until extinctions occur and feasibility of the remaining species is reached (*Details*: “Materials and methods”). Examples of several invasion attempts are shown in Fig. 2.

**Figure 1:**
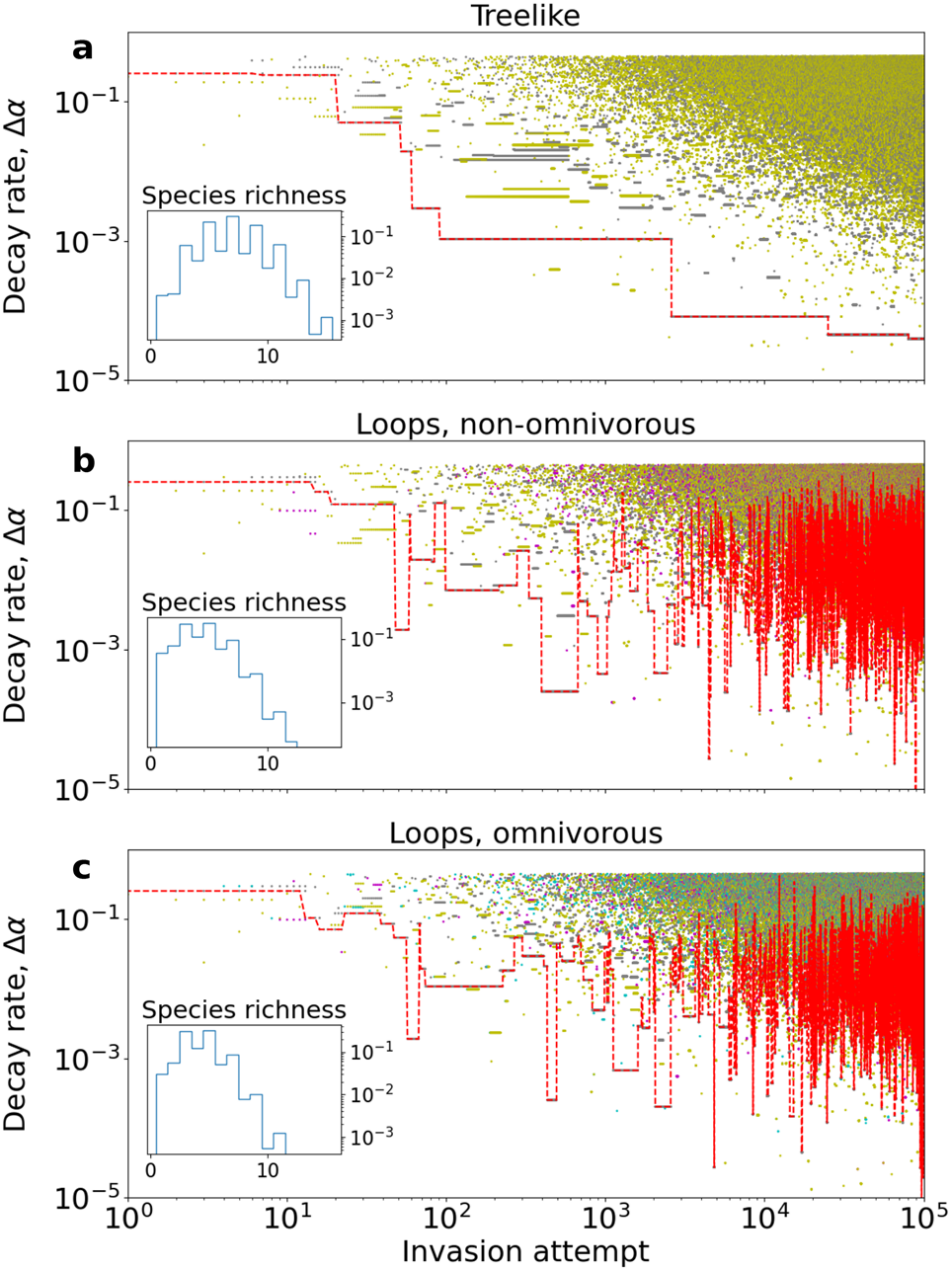
Evolution of three food webs using different assembly rules. All main panels show decay rates of all species present plotted against invasion attempts, that is, evolutionary steps. The decay rates are plotted as $\Delta \alpha \equiv \alpha - \alpha _{min}$, where $\alpha _{min}$ denotes the lower limit on decay rates (*compare*: Table 1). The thin red line highlights the currently lowest producer decay rate. Grey symbols denote producers, yellow and magenta symbols denote consumers of one or two resources, respectively. Cyan symbols denote omnivores. (**a**) Food webs where only one resource per consumer is allowed, yielding a treelike food web without loops. (**b**) Consumers can have either one or two resources at the same trophic level. (**c**) Consumers are allowed one or two resources at any trophic level $l\ge 1$. Note that both axes use logarithmic scaling. Insets: Normalised histograms of species richness, using all data. Note the logarithmic vertical axis scaling.

**Figure 2:**
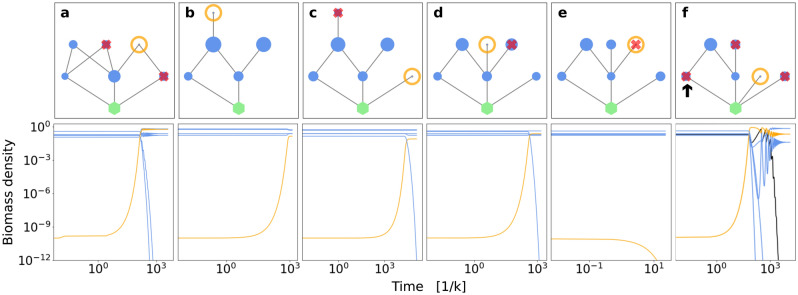
Time series of a food web during several invasions. The panels (**a**–**f**) respectively correspond to invasion attempts 40312–40314, and 40316–40318 in Fig. 1c. *Upper row*: In each panel, orange circles and red “**x**”-symbols denote the invasive and extinct species, respectively. The vertical coordinate denotes trophic level, and node areas represent initial biomass densities. The green hexagon represents the basic nutrient source. (**a**) A species successfully invades the food web, but causes the extinction of two resident species, among these one of its own resources. (**b**) the invader is successful without causing any extinctions. (**c**) The invader is a primary producer and causes extinction of the invader from (**b**). (**d**) The invader replaces a resident species of same niche as the invader. (**e**) The invader is unsuccessful in invading the food web as it shares a niche with one of the resident species. (**f**) The invader is a primary producer and causes the extinction of three resident species, among these the primary producer with lowest decay rate, corresponding to largest intrinsic fitness, which is highlighted by the black arrow. *Lower row*: Time series corresponding to each of the food webs above, where time is measured in units of the inverse primary producer growth rate, $k^{-1}$. Blue and orange lines represent resident and invasive species, respectively, as the new steady state is approached. The black line in the last panel represents the producer with lowest decay rate. Note the double-log axis scaling.

### Loops profoundly impact food web evolution

To make sure our results do not depend on the details of the invasion process we allow for several qualitatively distinct evolutionary processes: (i) treelike food webs, where each consumer has a single resource; (ii) non-omnivorous food webs with loops; (iii) omnivorous food webs. Loops are known to be relevant for sustained limit cycles and chaotic attractors, thus widening the range of dynamical properties. Indeed, we find treelike food webs to stand out in that fitness, measured by species decay rates, indefinitely increases in the evolutionary process (Fig. 1a, dotted red line), a finding consistent with the recent literature^30^. This indefinite fitness improvement hinges on the absence of network loops: a given primary producer can only be replaced by an invading primary producer of greater intrinsic fitness, that is, lower decay rate.

Allowing for network loops, evolved food web do not show indefinite fitness improvement (Fig. 1b,c) and mean species richness somewhat decreases (Fig. 1, insets). All histograms show a systematic difference in odd and even species richness, with food webs of odd species richness being the most frequent. This tendency is most pronounced for treelike food webs. We interpret this as a manifestation of the requirement of non-overlapping pairing^28^. Treelike food webs are feasible and stable if every species in the food web can be coincidentally paired with a connected species or nutrient that is not part of another pairing. In food webs of even species richness the nutrient is never included in such a pairing. Food webs consisting of several smaller trees that are connected through the nutrient source are therefore only feasible if every tree satisfies this requirement individually. On the contrary, the nutrient is always included in a pairing in food webs of odd species richness, and therefore odd food webs are more likely to be feasible. To a lesser extent this tendency is also found in the histograms representing food webs with network loops. We interpret this as resulting from the fact that 40-60% of the food webs from simulations allowing network loops are in fact treelike.

Why do loops counteract indefinite fitness improvement? This can be seen as a manifestation of relative, rather than absolute, fitness, where a species can consume two resources and thereby can help eliminate even primary producers of high intrinsic fitness (Fig. 1b,c). An example of this is illustrated in Fig. 2f), where the intrinsically fittest producer is a node in a food web loop, and is driven to extinction during the invasion of a producer with lower intrinsic fitness.

The evolution of intrinsic fitness in Fig. 1 implies that allowing for interaction loops makes resident species more vulnerable to extinction during invasions, because parameters that characterise high intrinsic fitness before an invasion might characterise low intrinsic fitness during the invasion. This is supported by the cumulative distribution of resident times (Fig. S1a), where residence times in food webs with network loops fall off faster than the residence times in treelike food webs. In Fig. S1b we observe that in accordance with this, the distribution of extinction event size falls off faster for treelike food webs (Fig. S1b), where the extinction event size is measured relative to the total number of species (species richness) in the food web. Fig. S1b therefore implies that interaction loops make food webs less robust to invasions, as invasive species tend to create larger extinction events here than in treelike food webs. Finally, we find invasive species to have higher success rates when invading food webs with interaction loops, and the success rate is found to increase with $\beta$. In simulations with $\beta =0.75$ we observe 11.5%, 27.2% and 29.8% for treelike, non-omnivorous, and omnivorous food webs with loops, respectively. The implications of this are twofold. On one hand, it is easier to assemble feasible food webs when multiple resources and omnivory are allowed. On the other hand, these food webs are more susceptible to invasions and their resident species are more vulnerable. If a food web contains two-resource species, removal of one of the two resources of a species $S_i$ by an invader can already lead to a cascading extinction of *S*, as exemplified by Fig. S2.

### Robustly bi-modal eigenvalue spectra

**Figure 3:**
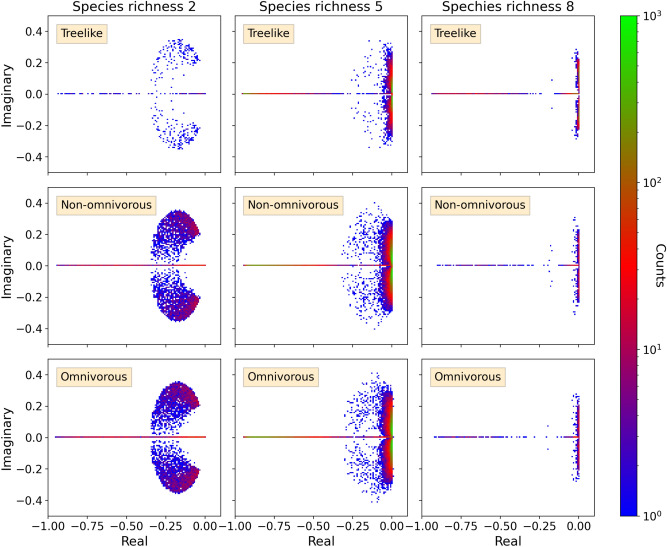
Complex eigenvalue spectra of evolved food webs. Each panel represents the two-dimensional histogram in the complex plane. Species richness and invasion mechanism are as labelled in panels, that is, rows of panels represent treelike, non-omnivorous, and omnivorous food webs. Note that the colour scale is logarithmic, with green marking the areas with largest likelihood of eigenvalues (Details: “Materials and methods”). Eigenvalue spectra of omnivorous food webs of other species richnesses can be seen in Fig. S3.

We now turn to the eigenvalue spectra of the evolved complex food webs, which we present as two-dimensional histograms in the complex plane (Fig. 3). Each simulation conducts $10^5$ invasion attempts, yet the number of unique feasible food webs is considerably lower, that is, approximately equal to the aforementioned rates of successful invasions. Furthermore, the number of unique feasibly food webs drastically decreases with species richness. While the data shown represent relatively small networks, we find that key spectral features are very systematic as function of species richness. A generic feature is that spectra typically have many eigenvalues with small negative real parts. Further, the real parts scatter more and more closely at small negative values, as species richness increases beyond two. All spectra contain a considerable fraction of purely real eigenvalues, typically making up 15–30% of a spectrum.

### The origin of purely real eigenvalues

The first column in Fig. 3 represents food webs with species richness two. These simple food webs only have one feasible configuration, namely that of one primary producer and one consumer. Any differences between spectra in the left column are therefore purely statistical. These food webs can be considered as isolated interactions between a consumer and its resource, hence the analytical eigenvalues of this food web can provide some insight on the dynamics underlying the eigenvalue spectra. From the analytical eigenvalues we obtain that an eigenvalue is purely real if the inequality

3 $$\begin{aligned} \beta \eta \le \frac{1}{2}\left( \gamma + \sqrt{\gamma ^2 + k\gamma }\right) , \,\,\,\,\,\,\,\,\text {with}\,\, \gamma \equiv \frac{\alpha _2}{1-\alpha _1/k}, \end{aligned}$$

is fulfilled (*Details*: Sec. S3). Here, $\alpha _1$ and $\alpha _2$ are the decay rates of the resource and the consumer, respectively, and $\beta \eta$ is short for $\beta _{21}\eta _{21}$, the “consumption rate” of the consumer. $\gamma$ can be interpreted as the inverse intrinsic fitness of the food web.

From feasibility, we have the additional requirement of $\gamma < \beta \eta$, hence, the consumer’s “consumption rate” is bounded also from below. As *k* decreases, the lower and upper boundaries on $\beta \eta$ approach one-another until they are equal for $k=0$. A food web with low producer growth rate is therefore likely to have complex eigenvalues. In the opposite limit, when $k\rightarrow \infty$, or equivalently $\alpha _1 \rightarrow 0$, we see that $\gamma$ reduces to $\alpha _2$. In the first limit Eq. (3) reduces to $\beta \eta \le \infty$ which will always be satisfied and all eigenvalues are therefore purely real in this limit. This corresponds to a food web where the consumer has infinite access to resources and there is no stress or constraints on the web that could cause oscillations. In the limit where $\alpha _1 \rightarrow 0$, the eigenvalues pick up an imaginary component when $\beta \eta$ is large compared to $\alpha _2$ and *k*. This occurs when the consumer population has a large intrinsic growth rate, thus heavily exploiting its resource.

Overall, purely real eigenvalues characterise food webs where consumption of the resource is moderate compared to the intrinsic fitness of the resource. This corresponds to an over-damped limit where the consumer does not consume enough to cause any significant displacement of the resource population, hence a perturbation of the consumer population will not spread to its resource. For higher species richness the Jacobian quickly becomes too complicated to be solved analytically. Even so, we expect the dynamics between a consumer and its resources to be conceptually analogous, namely that “sustainable over-consumption” yields oscillating densities and complex eigenvalues.

The set of smallest and largest real-valued eigenvalues is obtained when $\beta \eta$ is only slightly larger than $\gamma$, hence barely satisfying the criterion of feasibility. The eigenvalues then reduce to $\lambda _{\pm } = -\frac{k-\alpha _1}{2} \pm \frac{k-\alpha _1}{2}$. $\lambda _+$ is always zero, that is, food webs of species richness two are always stable, and with our choice of parameters $\lambda _- \ge -0.95$. We observe approximately the same range of real values in all numerical spectra of any species richness, thereby implying that the choice of parameters might be more important for the spectrum width than the structure of the food web.

The overall shape is qualitatively similar for all food web structures (*see*: Fig. 3). Importantly, omnivorous spectra are the only ones to contain also eigenvalues with positive real part, that is, unstable eigenvalues. These food webs do therefore not converge to their equilibrium state after an invasion, but are displaying periodic or chaotic dynamics (*Details*: “Materials and methods”). The unstable eigenvalues are all barely larger than zero, hence hardly visible in Fig. 3. Interestingly, non-omnivorous food webs with network loops exhibit the same species richness and approximate connectivity as the omnivorous food webs, yet they do not yield unstable eigenvalues. The differences between treelike food webs and food webs with network loops discussed earlier must therefore be unrelated to the stability of the food webs, thus emphasising the difference between stability to perturbation of a given food web and its robustness to invasions. For omnivorous food webs the fraction of unstable eigenvalues increases with species richness and decreases with $\beta$. Intuitively, it seems reasonable that there is a relation between instability and low consumption efficiency. A species with a low consumption efficiency has to compensate by consuming more biomass, thereby putting more stress on its resources. Only for $\beta =1$ are there no unstable omnivorous eigenvalues.

**Figure 4:**
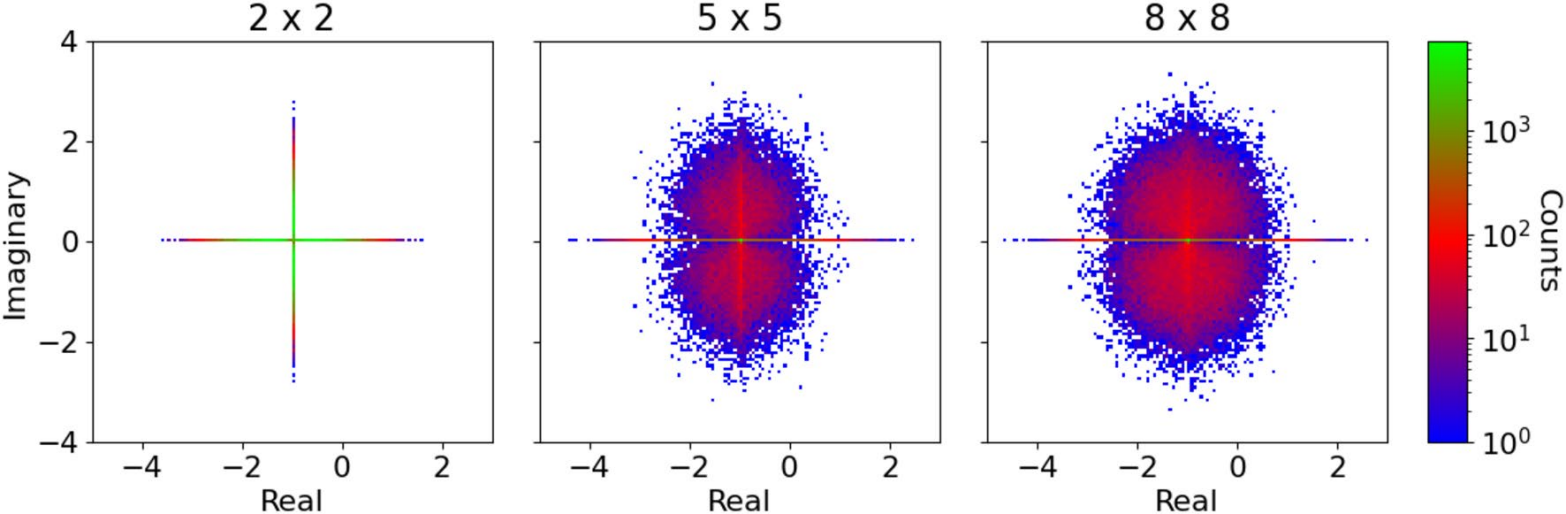
Complex eigenvalue spectra of random matrices. Heat maps of eigenvalue spectra of random matrices, corresponding to the respective species richnesses shown in Fig. 3. Off-diagonal entries are drawn from a normal distribution with probability $p(N) = \frac{N^2+21N-28}{9N(N-1)}$ (*Details*: Sec. S5), and are otherwise set to zero. Diagonal elements are set to $-1$.

We now compare the evolved spectra (Fig. 3) to their random counterparts (Fig. 4). The diagonal entries represent self regulation of each species and are set to $d = -1$. Off-diagonal entries are drawn from ${\mathcal {N}}(0, 1)$ with probability *p*(*N*), and are otherwise 0.

4 $$\begin{aligned} p(N) = \frac{N^2+21N-28}{9N(N-1)}, ~~\text {for } N>1, \end{aligned}$$

where *N* is “species richness”, that is, the number of rows (or columns) of the matrix. This corresponds to the implemented connectivity in the simulation allowing network loops and omnivory, that is, the connectivity of omnivorous food webs given no extinctions occur (*Details*: Sec. S5). As predicted by spectral theory of random matrices, the spectra are centred around *d* on the real axis and approach a circular geometry as the size of the matrix increases. Already for $N=2$ does the spectrum contain unstable eigenvalues. The fraction of unstable eigenvalues increases with *N* as the circle radius increases. Also for random spectra do we observe a large fraction of purely real eigenvalues. We attribute this to the small size of the matrices, being much smaller than the infinity limit for which the law was derived^40^.

**Figure 5:**
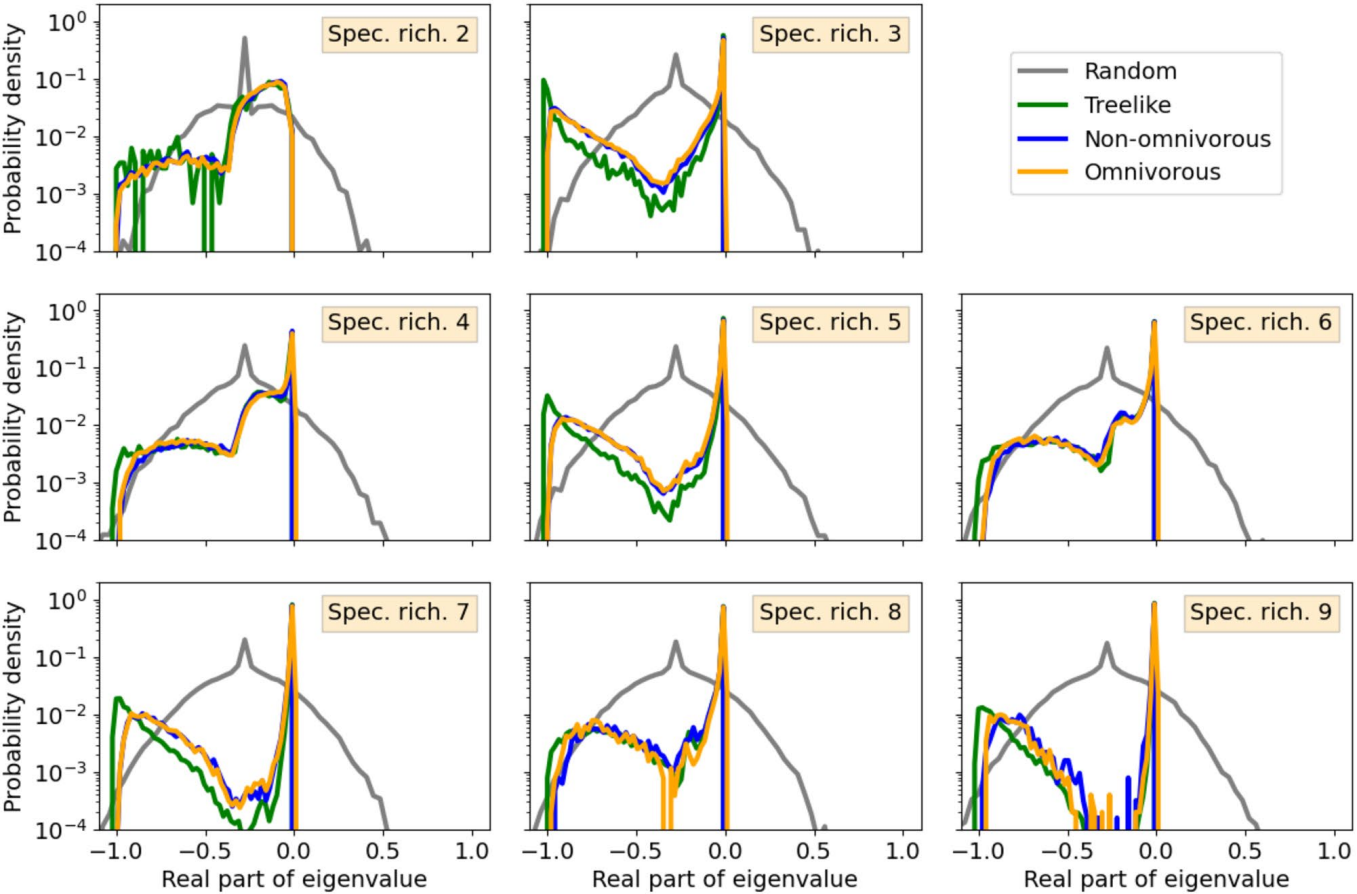
Distribution of eigenvalues along the real axis. Normalised frequency distributions of eigenvalues along the real axis for all food web structures and random matrices for species richness $2-9$. Eigenvalues representing food webs are taken from simulations using a range of values of $\beta$ (Table 1), since varying $\beta$ does not have significant effects on the real-part distributions (*Details:* Sec. S6). All distributions are scaled to start in $-1$. Note the logarithmic vertical axis scaling.

Finally, we study the real-part frequency distributions of eigenvalues of all four types (treelike, non-omnivorous, omnivorous and random). The frequency distributions for species richness 2–9 can be seen in Fig. 5, where each distribution consists of data from various values of $\beta$ (see Table 1). In order to facilitate comparison of the functional form of the frequency distributions, rather than the range, the frequency distributions are scaled to be bounded by $-1$ on the real axis, that is, we divide each data point by $(|\min \{x\}|)^{-1}$ where *x* is the data points of the distribution. Frequency distributions representing the evolved food webs follow approximately the same curve for a given species richness, and are distinctively different from the random matrices. As also seen in Fig. 3 omnivorous distributions are the only to extended to positive values for species richness greater than two.

Once again, we observe quantitative differences between food webs with odd and even species richness: For odd species richness the distribution is bi-modal with a global maximum near $x=0$ and a secondary maximum near the lower limit, that is $x=-1$. For even species richness, the distribution is initially less strongly peaked. Yet, as species richness increases, a sharp peak emerges around $x = 0$. The distribution thus becomes more similar to that of the food webs with an odd number of species.

The intermediate part of the spectrum is increasingly depleted of eigenvalues at higher species richness. Comparing Fig. 5 with Fig. 3 we see that the left part of all distributions consists of purely real eigenvalues, whereas it is mostly complex eigenvalues that make up the global maximum near $x=0$. This implies that perturbations can be divided into two main groups: perturbations from which the food web quickly returns to the respective steady state, and perturbations that induce oscillations from which the food web takes very long to recover. The peak consisting of purely real eigenvalues near $x=-1$ does not change notably with species richness, indicating that, independent of species richness, food webs are robust to certain perturbations. In accordance with this we observe that food webs of all species richness usually return quickly to their steady states *after* an unsuccessful invasive species goes extinct. The main peak (near $x=0$) becomes both higher and narrower with increasing species richness, that is, the food webs become quasi-stable. In larger food webs there are more species that can be disturbed by a perturbation, which might prolong the effect of the perturbation, that is, push eigenvalues towards zero on the real axis. Overall, we thus find that the histogram of complex food webs becomes strongly bi-modal as food webs consisting of many species are approached in an evolutionary process, whereas random matrix spectra are consistently uni-modal. In Sec. S8–S9 we consider the robustness of the results in Fig. 5 by varying the parameter distributions and implementing Holling type-II response, respectively.

## Conclusion

Using the established generalised Lotka–Volterra equations, we have evolved synthetic food webs through successive invasion attempts by random species. This evolutionary process followed simple assembly rules, yielding three different food web structures: ’treelike’ without network loops, ’non-omnivorous’ allowing network loops between strict trophic levels, and ’omnivorous’ with network loops and omnivory allowed. Data from ’treelike’ food webs reproduced known results for this food web structure^30^. We found that when allowing network loops in food webs, the notion of ’absolute fitness’ does no longer apply. Food webs with network loops are thus not able to systematically improve fitness with evolution, thus yielding a qualitatively different evolutionary process. We observed both shorter resident times and larger extinction events in food webs with network loops, than in their treelike counterparts. Despite these qualitative differences in the evolutionary process, all resulting spectra have similar features, with pronounced bi-modal real part histograms, and increasing species richness sharpening the bi-modality. By contrast, random matrix spectra remain uni-modal and, by construction, show no structural changes when species richness is increased.

Our approach uses a canonical set of equations for consumer-resource interactions, often taken as a simple starting point for the theoretical study of complex food webs. Yet, many variants exist, which could allow for greater realism or additional routes to coexistence. For example, Holling type-II functional responses may be a means of widening the range of coexistence, thus likely increasing achievable species richness. It could further be insightful to explore the effects of mutualism^20^, parasitism^19^, or the detritus network^41^ on opening further pathways to coexistence. It should then be explored if any of these additions bring the community matrix spectra closer to the spherical geometries typical of random matrices.

## Materials and methods

**Figure 6:**
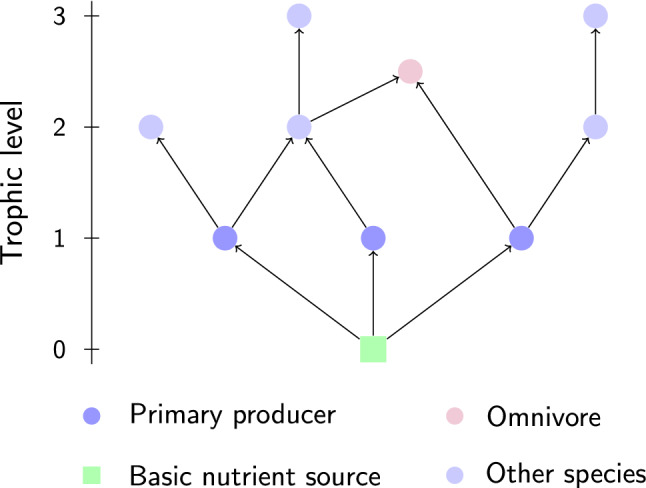
Example of an evolved food web. Species types as indicated in the legend. The vertical axis marks the trophic level, *l*. A species’ trophic position is defined using average food chain length.

### Definitions

We here define a *food web* as the directed network representing all energy fluxes in an ecosystem. Vertices and edges represent species (which can be consumers or resources or both) and their interactions, respectively. An example (Fig. 6) highlights a basic physical or chemical nutrient source, such as solar radiation or sugar, primary producers (autotrophs), such as plants, their consumers, and omnivores. We here measure a species’ trophic level, *l*, as the weighted mean of its resources’ trophic levels incremented by one. Primary producers are distinct in that they only consume basic nutrient sources, hence they always have $l = 1$. An *invader* is defined as a species previously not present in an existing food web, which may reach a feasible steady state when initialised at an infinitesimally small initial biomass concentration and exposed to the existing food web.

### Stability

The RHSs of Eqs. (1) and (2) are linear in the species populations $S_i$, therefore we can obtain the steady state of species concentrations from the matrix equation^28^

5 $$\begin{aligned} \mathbf {S^*} = {\mathbf {R}}^{-1}\cdot {\mathbf {K}}\,. \end{aligned}$$

Here, $\mathbf {S^*}$ is the vector of the species’ steady state populations, ${\mathbf {R}}$ is the matrix containing all interaction coefficients, and ${\mathbf {K}}$ contains the species specific growth and decay rates. From Eq. (5) it follows that food webs with invertible interaction matrices have unique steady states^42^. Feasibility of the food web additionally requires that all $\mathbf {S^*}$ be positive, a point we return to below.

For small perturbations away from equilibrium, linear stability of the steady states can be determined from the eigenvalues of the community matrix, ${\mathbf {C}}$, that is, the Jacobian of the Lotka–Volterra equations evaluated at the fixed points, namely

6 $$\begin{aligned} C_{ij} \equiv \frac{\partial }{\partial S_j}\dot{S_i}\left( {\mathbf {S}}(t)\right) \Bigr |_{{\mathbf {S}}={\mathbf {S}}^*}. \end{aligned}$$

Here $S_j$ and $\dot{S_j}\equiv \partial S_j/\partial t$ are the species biomass concentrations and their time derivatives, respectively, *compare* Eqs. (1) and (2), and $\mathbf {S^*}$ is the steady state species vector, *see* Eq. (5). ${\mathbf {C}}$ is asymmetric, partly stemming from the asymmetry with regard to what the predator gains and what the prey loses. The general community matrix is written out explicitly in Sec. S10. The food web is linearly stable if all eigenvalues of the community matrix ${\mathbf {C}}$ have negative real parts, corresponding to local attraction towards the steady state^43^.

Previously, it has been suggested that the community matrix can be represented by a random matrix^8,9,44^. The eigenvalue spectrum of a random matrix scatters within a semicircle as the size of the matrix tends towards infinity. The radius of the semicircle increases with size and density of the random matrix^45^. If a large and complex food web can accurately be modelled by a random matrix, it is therefore very likely to yield at least one eigenvalue with positive real value, hence to be unstable.

### Computer simulations

#### Algorithm

Simulations are performed in C++, using the Eigen library^46^ to perform the matrix operations described in the previous section. The simulation builds food webs by successively adding new species to the existing food web. Once disrupted, one or more species might go extinct while the food web settles in its new steady state. Each simulation conducts $10^5$ invasion attempts to the initial food web consisting of a basic nutrient source and a primary producer feeding on the source. A pseudo-code describing the simulation is shown in Algorithm 1.
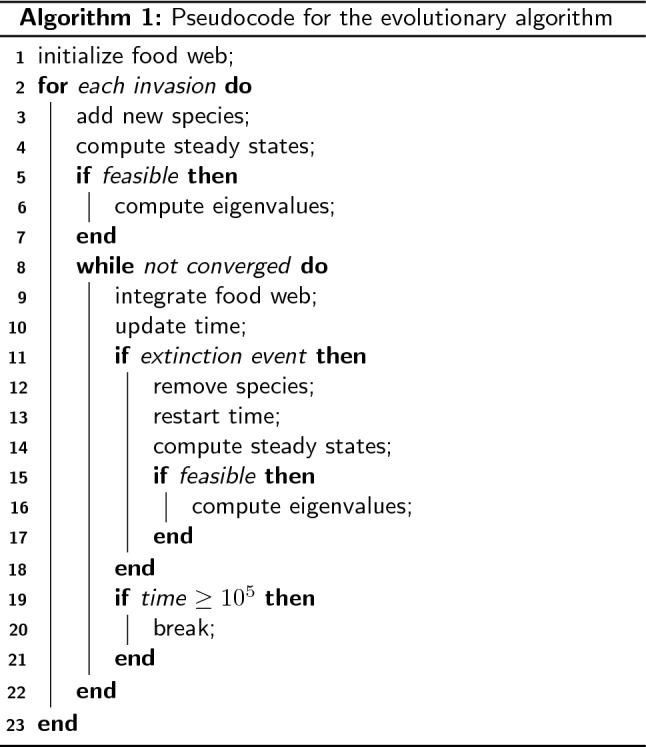


With each invasion $\mathbf {S^*}$ is computed. If the food web is feasible, that is, if $S_i > 10^{-14}$ for all *i*, the eigenvalues of the community matrix are also computed. At every time step the food web is checked for extinct species. A species, $S_i$, is considered extinct if $S_i \le 10^{-12}$. Such a species is subsequently removed from the food web by setting its biomass density and all other parameters to zero, including links to resources/consumers. After every extinction the steady states and community eigenvalues are recomputed since they will have changed. For both physical and numerical reasons it is meaningful to set the extinction threshold larger than zero. However, this means that extinctions can occur even in food webs that are theoretically stable.

#### Numerical convergence criteria

A food web is considered to have converged to its steady state when all species in the food web satisfy $|S_i - S_i^*|/S_i^* < 10^{-6}$ for all *i*. For time efficiency this criterion is softened to $10^{-2}$ if the food web does not converge within $t = 10^{4}~\text {k}^{-1}$. Furthermore, if the food web still has not converged at $t = 10^5~\text {k}^{-1}$, the integration is broken and the food web is considered to be “non-convergent”. The dynamics of the non-convergent food webs are not investigated further, and might be periodic, chaotic or even converging at a very low rate.

We only apply the two first convergence criteria to food webs that are feasible and linearly stable, since these are the only food webs we expect to converge. We assume linearly stable food webs that do not converge within the given range of *t* to be in fact converging, yet too slowly to be detected by our naïve test for convergence. We therefore set the final biomass densities of linearly stable, non-convergent food webs equal to the steady states, that is we set $S_i(t = 10^5 ~k^{-1}) = S_i^*$. The few food webs that are feasible, but not linearly stable are integrated until $t = 10^5~\text {k}^{-1}$, or until an extinction occurs. If a species in the food web goes extinct, both the feasibility and linear stability of the food web are recomputed, and the time is restarted. Unfeasible food webs are always integrated until one or several extinction events renders them feasible. Following this procedure, invasive species are only added to feasible food webs long after previous invasions or extinctions.

#### Declaring a species

In the simulations we use objects to represent each species. A class ’species’ is created, with the derived class ’producer’ containing the subgroup of primary producers (See Sec. S7 for a class diagram.). Each member of species (hence also of producer) contains a biomass density variable, species- and link-specific parameters and a trophic level, corresponding to $S_i, k_i, \alpha _i, \eta _{ji}, \beta _{km}$ and $l_i$ from Eq. (1) and (2). When a species is declared, it is assigned a small initial biomass density, $S_i(t=0) = 10^{-10}$, and the intrinsic parameters $\alpha$ and *k*. For simplicity $k_i = 1$ for all primary producers. $\beta$ is fixed for all species within one simulation, but varied between simulations (See Table 1). For all species $\alpha$ is drawn from ${\mathcal {U}}(0.05, 0.5)$. The interaction parameters and trophic levels depend on the structure of the specific food web, and will be assigned to a species as it is added to the food web. The invasion ratio of producers to species is set to 1:2. Parameters and corresponding distributions are collected in Table 1.

**Table 1:** Simulation parameters and their sample distributions.

Parameter	Distribution
*k*, growth rate	1
$\alpha$, decay rate	${\mathcal {U}}(0.05, 0.5)$
$\eta$, interaction rate	${\mathcal {U}}(0.01, 1)$
$\beta$, reproduction efficiency	0.25, 0.5, 0.75, 0.9, 1

#### Modelling invasion attempts

An invader is added to the food web when an interaction link is established to one or several of the resident species. An invasive primary producer is connected by a single consumption link to the basic nutrient source, whereas invasive species at higher trophic positions are assigned at least one resource species. In simulations, where the species are allowed to consume more than one resource, in effect allowing for *network loops*, a second link is established with a fixed probability. In simulations, where strict trophic levels are demanded, that is, omnivory is not allowed, we only accept the randomly selected second resource if it has the same trophic level as the first. In the omnivorous case, the second resource is always accepted, thus leading to a blend of species with single consumption links, two links to resources and the same trophic level, and two links to resources at distinct trophic levels. To roughly compensate for the lower acceptance rate of the second consumption link in the non-omnivorous case, the probability of adding a second resource in a non-omnivorous food web is set to 1.5*p* where $p = 1/2$ is the probability of a second resource in an omnivorous web. When an interaction is created $\eta _{ki}$ is drawn uniformly from $\eta \in {\mathcal {U}}(0.01, 1)$. Finally, all species’ trophic levels are updated, reflecting the structure of the current food web.

Although it can be argued that a species would pick its resources after abundance or would specialise on a certain type of resource species^15^, this is not imitated in the current simulation. We argue that such additional constraints would further reduce the randomness of interactions. Our intention here is to compare an evolved food web’s community matrix with a that of a random counterpart—we hence aim to limit further restrictions or assumption on the types of interactions.

## Supplementary information

Supplementary Information.

## Data Availability

Not applicable. The program code is available in a GitHub repository: https://www.github.com/siljabl/FoodWebs. The simulation data used in this manuscript can be reproduced using the code attached (“Materials and methods”).
