# Supplementary material for: The geometry of evolved community matrix spectra
Source: Sci Rep. 2022 Aug 29;12:14668. doi: 10.1038/s41598-022-17379-6 (PMC9530164; doi:10.1038/s41598-022-17379-6)
Supplement: Supplementary file 1 — Supplementary Information. [file 41598_2022_17379_MOESM1_ESM.pdf]

Supplementary Information

S1 Distributions of residence time and extinction event sizes

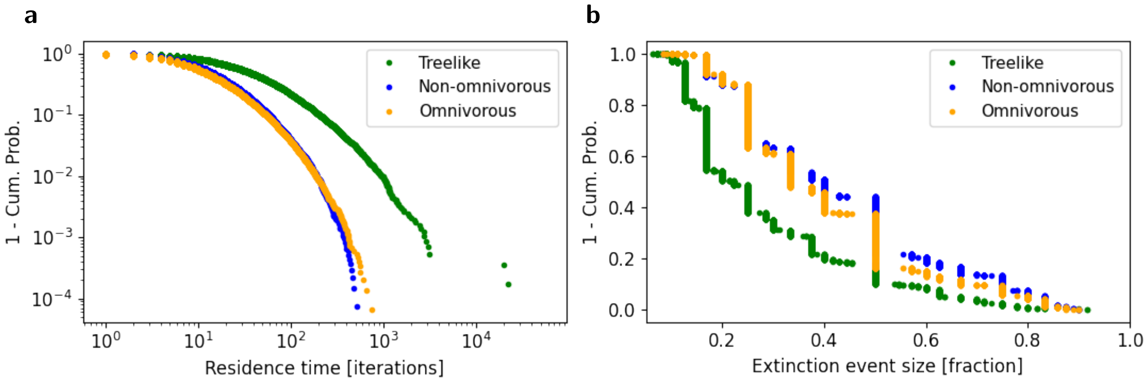

Figure S1: **Vulnerability of resident species in three types of food webs.** **a**, Cumulative probability distribution of residence times. **b**, Cumulative probability distribution of extinction event size.

Resident times (Fig. S1a) and fractional extinction event sizes (Fig. S1b) of three different food web types. Resident times in all three food webs fall off approximately like $\propto \exp\left(-bt^{1/c}\right)$, though less accurately for large t. For the treelike food web we observe $b \approx 11$ and $c \approx 9$, whereas for the two food webs with network loops we observe $b \approx 12.5$ and $c \approx 8$.

S2 Example of extinction cascade

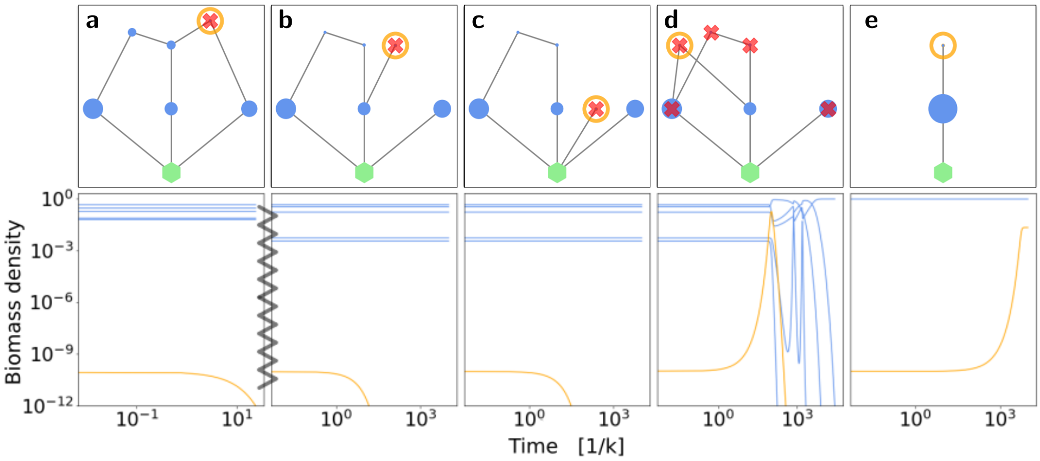

Figure S2: **Example of extinction cascade. a–e,** Five invasion attempts in an omnivorous food web with $\beta = 0.75$. Presentation and labelling analogous to that in Fig. 2. The figures correspond to the invasion attempts 76901, 76913–76916. **a**, Unsuccessful invasion attempt by omnivorous invader; **b**, Unsuccessful invasion attempt by consumer of one resource; **c**, Unsuccessful invasion attempt by primary producer, **d**, The invader causes an extinction cascade killing itself along with four resident species; **e**, Successful invasion by a species consuming the only resident species in the food web.

S3 Analytical spectrum of food webs with two species

The only feasible food web with two species is that of one producer and one species consuming the producer. The steady states of this food web are

$$S_1^* = \frac{\alpha_2}{\beta\eta}, \qquad S_2^* = \frac{k}{\eta}\left(1 - \frac{\alpha_2}{\beta\eta}\right) - \frac{\alpha_1}{\eta} \tag{S1}$$

for the producer and consumer, respectively. Here $\beta = \beta_{21}$ and $\eta = \eta_{21}$. Inserting this in Eq. (6) and diagonalising yields the eigenvalues

$$\lambda_{\pm} = -\frac{k\alpha_2}{2\beta\eta} \pm \sqrt{\left(\frac{k\alpha_2}{2\beta\eta}\right)^2 + \frac{k\alpha_2^2}{\beta\eta} - (k - \alpha_1)\,\alpha_2}. \tag{S2}$$

The food web is stable if the real parts of all eigenvalues are negative. The real part of $\mathrm{Re}(\lambda_-)$ is always negative. $\mathrm{Re}(\lambda_+)$ is only *non-negative* if the square root is real and equal to or greater than $\frac{k\alpha_2}{2\beta\eta}$. After some basic algebraic operations this criterion reduces to the following restriction on the species parameters

$$1 < \frac{\alpha_1}{k} + \frac{\alpha_2}{\beta\eta}. \tag{S3}$$

Eq. (S3) is further restricted by feasibility of the food web, which requires the steady states of Eq. (S1) to be positive. S_1^* is always positive, but S_2^* is only positive if $\beta\eta > \frac{\alpha_2}{1-\alpha_1/k}$. Inserting this in Eq. (S3) now yields

$$1 < \frac{\alpha_1}{k} + \frac{\alpha_2}{\beta\eta} < \frac{\alpha_1}{k} + \frac{\alpha_2}{\alpha_2}\left(1 - \frac{\alpha_1}{k}\right) = 1. \tag{S4}$$

This condition can never be satisfied and consequently a feasible food web with two species will always be stable.

The eigenvalues are purely real if the square root of Eq. (S3) is bigger than or equal to zero. Again using some basic algebra this criterion transforms to

$$(\beta\eta)^2 - \gamma\beta\eta - 0.25\gamma k \leq 0, \qquad \text{with } \gamma \equiv \frac{\alpha_2}{1 - \alpha_1/k}. \tag{S5}$$

This second order polynomial is zero when

$$(\beta\eta)_{\pm} = \frac{1}{2}\left(\gamma \pm \sqrt{\gamma^2 + k\gamma}\right), \tag{S6}$$

and negative in the interval between the two roots. $(\beta\eta)_-$ is always negative, since $k\gamma > 0$ for all $\alpha_1 < k$. This root is not physically meaningful. Accordingly, the eigenvalues are purely real if and only if $\beta\eta \leq (\beta\eta)_+$.

S4 Omnivorous eigenvalue spectra of species richness 2–10

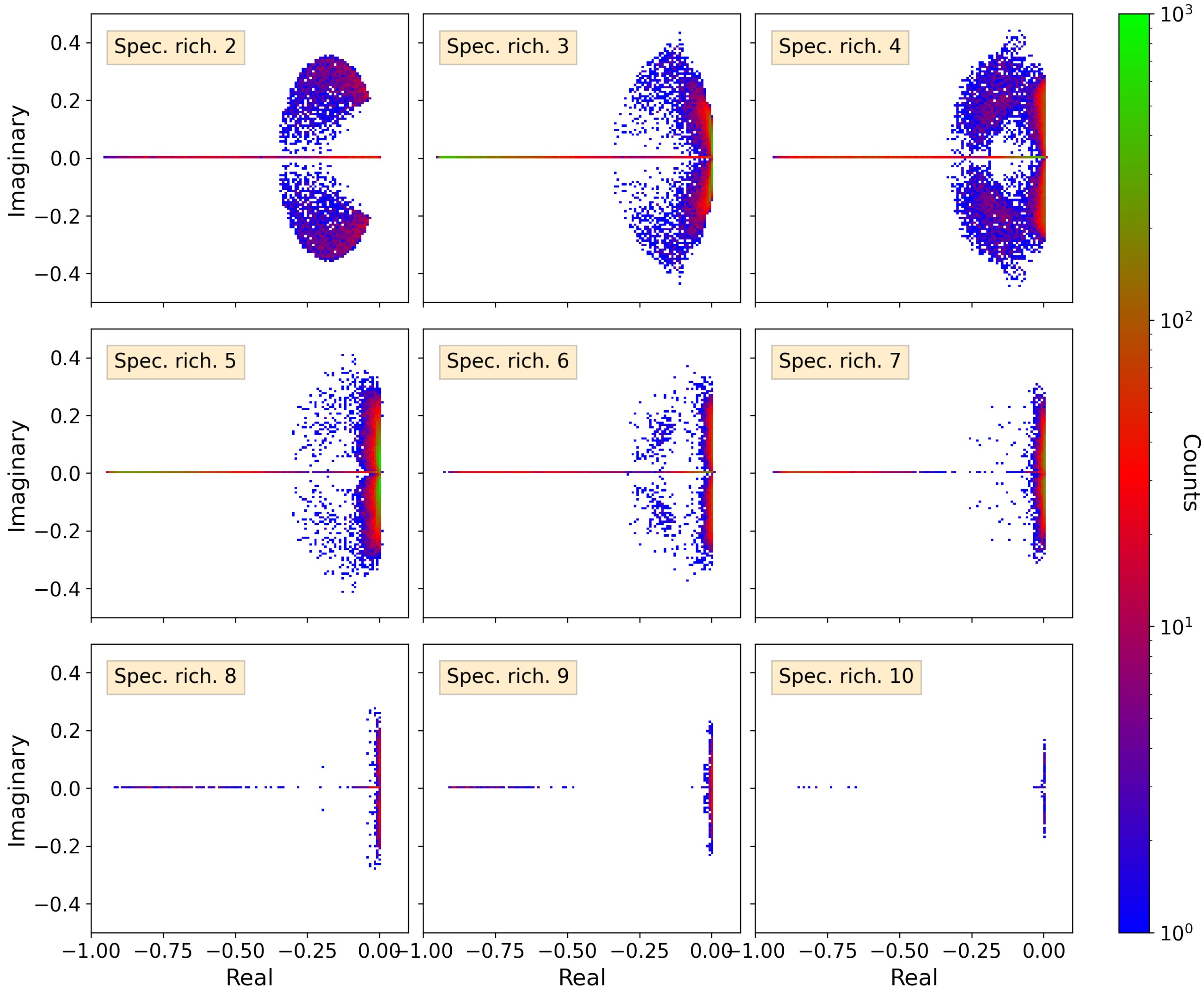

Figure S3: **Complex eigenvalue spectra of evolved omnivorous food webs with $\beta = 0.75$.** Each panel represents the two-dimensional histogram in the complex plane. Species richness as labelled in panels. Note that the colour scale is logarithmic, with green marking the areas with largest likelihood of eigenvalues. Left row corresponds to the omnivorous food webs in Fig. 3. As discussed in the main text, changing the invasion mechanics does not affect the spectrum notably.

Raw moment of n_i	Formula
$\langle n_i \rangle$	Np_i
$\langle n_i^2 \rangle$	$Np_i(1-p_i) + N^2 p_i^2$
$\langle n_i^3 \rangle$	$Np_i + 3N(N-1)p^2 + N(N-1)(N-2)p_i^3$

Table S2: The first three raw moments of the Binomial distribution, $n_i \sim B(N, p_i)$. Adapted from [2]

S5 Deriving the connectivity of omnivorous food webs

In this section we derive Eq. (4), that is the connectivity of omnivorous food webs as a function of number of species in the food web, N, given no extinctions occur. The connectivity of a food web is the total number of interaction links in the food web, multiplied by two (since every interaction appears twice in the community matrix), and divided by the total number of off-diagonal elements, $N(N-1)$. Here, we disregard the diagonal of the community matrix since it represents self-regulation and is set to $-d$ in the random matrix [1].

There are three types of species in an omnivorous food web in terms of number of interaction links: *primary producers*, with links to all other primary producers in the food web, *consumers with one resource* (1 link), and *consumers with two resources* (2 links). In addition, any of the three can be the resource of another species. To obtain the average number of interaction links stemming from each species type, we need to consider all possible configurations of the food web.

Starting with the primary producers, we compute the average number of producer links, L_p

$$L_p = \sum_{n_p=1}^{N} n_p(n_p-1)\binom{N-1}{n_p-1}\left(\frac{1}{3}\right)^{n_p-1}\left(1-\frac{1}{3}\right)^{N-n_p}. \tag{S7}$$

Here, n_p denotes the number of producers in the food web, and $n_p(n_p-1)$ is the number of producer links in a given food web. Since all food webs start with a producer, the position of the first producer is fixed and only $n_p - 1$ producers can be rearranged among $N-1$ species. For the same reason, the probability that an invasive species is a primary producer, $1/3$, is raised only to the power of $n_p - 1$. $\left(1-\frac{1}{3}\right) = 2/3$ is the probability that an invasive species is not a producer, i.e. is a consumer with one or two resources. We also need to distinguish the consumer types when we consider the different configurations of food webs with $N > 2$. However, this is already accounted for by the probability $2/3$. The probability of a specific configuration of consumers is $(1/3)^{N-n_p}$. The fact that any consumer can be replaced by the other consumer type without affecting the configuration of producers adds a factor 2^{N-n_p}.

We can rewrite Eq. (S7) as

$$L_p = \frac{3}{N}\sum_{n_p=0}^{N}\left(n_p^3 - n_p^2\right)\binom{N}{n_p}\left(\frac{1}{3}\right)^{n_p}\left(\frac{2}{3}\right)^{N-n_p}, \tag{S8}$$

and use that $\langle n_i^3\rangle - \langle n_i^2\rangle = 2N(N-1)p_i^2 + N(N-1)(N-2)p_i^3$ (See Tab. S2) to obtain

$$L_p = \frac{3}{N}\left[2N(N-1)\left(\frac{1}{3}\right)^2 + N(N-1)(N-2)\left(\frac{1}{3}\right)^3\right] = \frac{(N-1)(N+4)}{3^2}. \tag{S9}$$

Then we compute the average number of links of the consumer species, following mostly the same pattern as Eq. (S7). First we consider consumers of one resource, L_{c1}. Because the probability that the second species in the food web is a one-resource consumer is twice the probability that any other species is a one-resource consumer, we treat the two cases separately

$$\begin{aligned} L_{c1} = &\frac{2}{3}\sum_{n_c=1}^{N-1} 2n_c\binom{N-2}{n_c-1}\left(\frac{1}{3}\right)^{n_c-1}\left(1-\frac{1}{3}\right)^{N-1-n_c} \\ &+\frac{1}{3}\sum_{n_c=1}^{N-2} 2n_c\binom{N-2}{n_c}\left(\frac{1}{3}\right)^{n_c}\left(1-\frac{1}{3}\right)^{N-2-n_c}. \end{aligned} \tag{S10}$$

The first sum represents the case where the second species to be added to the food web is a consumer of one resource, whereas the second sum represents the case where the second species is a producer. The factor in front of each sum is the probability of each case, respectively. In both sums we sum over $2n_{c1}$ since each one-resource consumer contributes with one interaction, and every interaction appears twice in the community matrix. In the first sum the upper limit is $N-1$. Here, the position of one of the one-resource consumers is fixed, hence only $n_{c1}-1$ one-resource consumers can be rearranged among $N-2$ species. In the second sum the upper limit is $N-2$ because the two first species in the food web are producers, therefore n_{c1} one-resource consumers can be rearranged among $N-2$ species.

$1/3$ is the probability that an invasive species is a one-resource consumer in food webs of $N \geq 2$, and is therefore raised to $n_c - 1$ in the first sum and n_c in the second. As in Eq. (S7), we do not need to explicitly consider the different configurations of producers and two-resource consumers.

We rewrite Eq. (S10) as

$$\begin{aligned} L_{c1} = &\frac{4}{N-1} \sum_{n_c=0}^{N-1} n_c^2 \binom{N-1}{n_c} \left(\frac{1}{3}\right)^{n_c} \left(\frac{2}{3}\right)^{N-1-n_c} \\ &+ \frac{2}{3} \sum_{n_c=0}^{N-2} n_c \binom{N-2}{n_c} \left(\frac{1}{3}\right)^{n_c} \left(\frac{2}{3}\right)^{N-2-n_c}, \end{aligned} \tag{S11}$$

$$\Rightarrow L_{c1} = \frac{4}{N-1} \left(\frac{2(N-1)}{3^2} + \frac{(N-1)^2}{3^2} \right) + 2\frac{N-2}{3^2} = \frac{2N}{3}. \tag{S12}$$

Here, we have used Tab. S2 to replace the sums. Lastly, we compute the average number of links stemming from the consumer of two resources, L_{c2}

$$L_{c2} = \sum_{n_c=0}^{N-2} 2 \cdot 2n_c \binom{N-2}{n_c} \left(\frac{1}{3}\right)^{n_c} \left(\frac{2}{3}\right)^{N-2-n_c} = 4\frac{N-2}{3}. \tag{S13}$$

Where the sum only goes to $N-2$ since two-resource consumers are only added to food webs of $N \geq 2$. Finally, we combine Eq. (S9) and Eqs. (S12)–(S13) and divide by $N(N-1)$ to obtain the connectivity

$$\begin{aligned} p(N) &= \frac{(N-1)(N+4)}{3^2 N(N-1)} + \frac{2N}{3N(N-1)} + \frac{4N-8}{3N(N-1)}, \\ &= \frac{N^2 + 21N - 28}{9N(N-1)} \quad \text{for } N > 1. \end{aligned}$$

S6 The effect of β on eigenvalue spectra

Fig. 5 depicts the distributions of eigenvalues along the real axis combining all values of β. Here we therefore investigate how the choice of β affects the eigenvalue spectrum. In Fig. S4 histograms of eigenvalues along the real

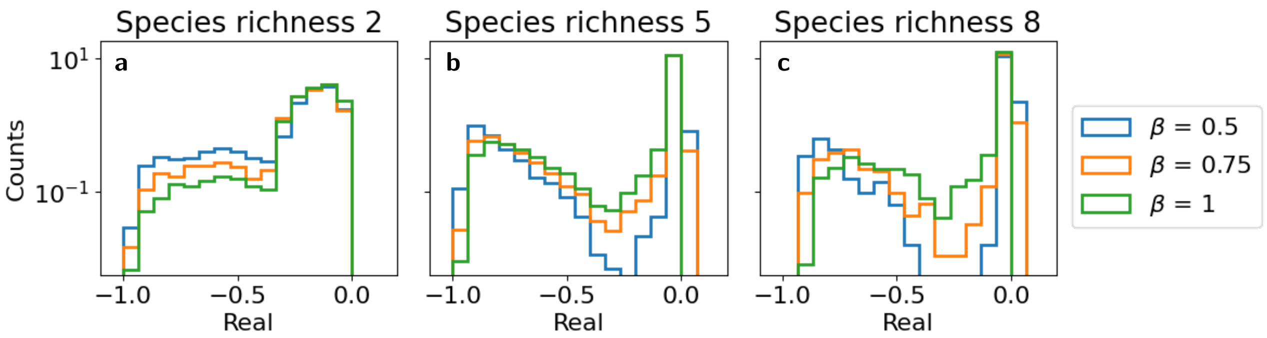

Figure S4: **Histograms of eigenvalues along the real axis for different β.** Normalised frequency histograms of eigenvalues along the real axis for omnivorous food webs.

axis are plotted for $\beta = 0.5, 0.75$ and 1. The range and overall shape are similar. For species richness two (Fig. S4a) the left plateau decreases in significance with increasing β. For larger species richness (Fig. S4b and c) the middle region is less depleted for high β, i.e. the spectrum become "less" biomdal with β. Even though varying β does affect the distribution of eigenvalues along the real axis, we still combine the distributions in Fig. 5 because they have peaks and dips in the same ranges of real values, and we are interested in comparing the overall shapes of the eigenvalue spectra, rather than the exact distributions.

Fig. S5 shows the corresponding histograms of eigenvalues along the imaginary axis. For all β and species richnesses

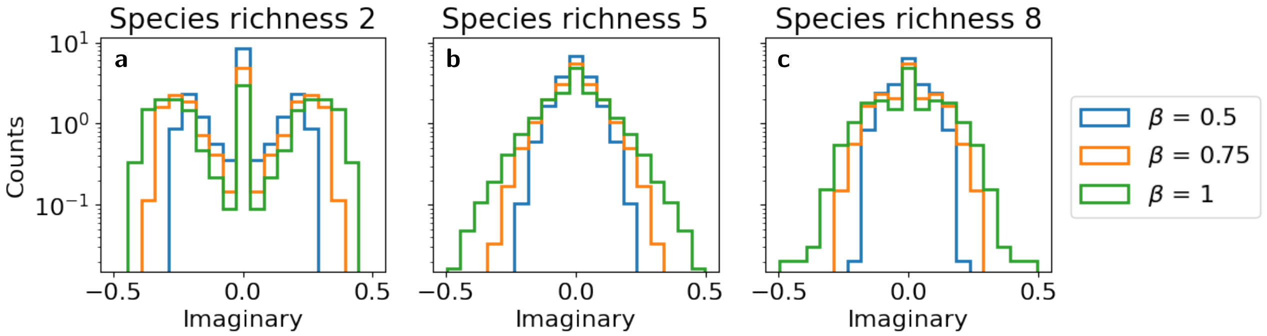

Figure S5: **Histograms of eigenvalues along the imaginary axis for different β.** Normalised frequency histograms of eigenvalues along the imaginary axis for omnivorous food webs.

there is a peak around 0 on the imaginary axis, as expected since most spectra contain a significant fraction of purely real eigenvalues. As β increases, so does the width of the imaginary distribution, whereas the overall shape remains the same.

S7 Class diagrams

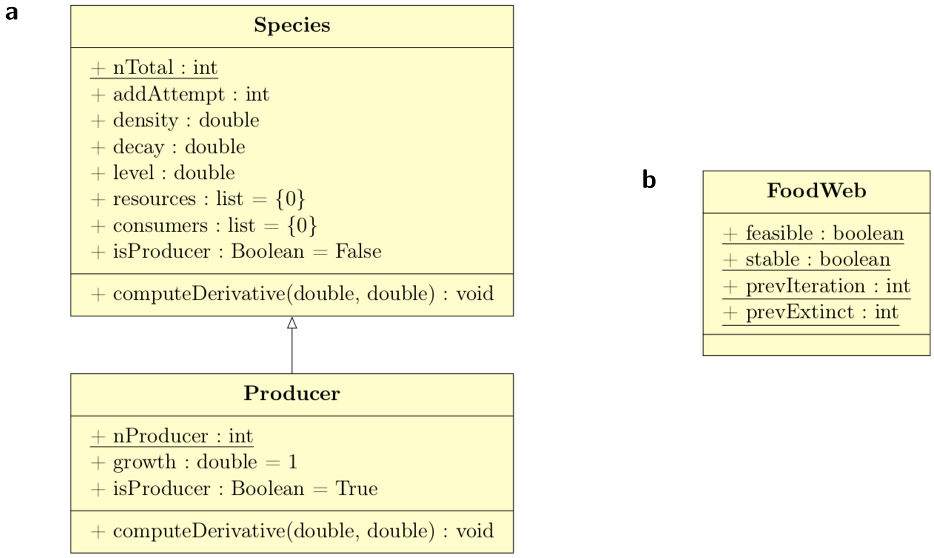

Figure S6: **Class diagrams from simulations.** **a**, The class Species and the derived class Producer. **b** The class FoodWeb.

Fig. S6a depicts the class diagrams of the class `Species` and the derived class `Producer`. `nTotal` is a global variable that keeps track of the total number of species in the food web. `addAttempt` is the iteration during which a given species invaded the food web. `density`, `decay` and `level` represent S_i, α_i and l_i, respectively. `resources` and `consumers` contain interaction parameters w.r.t. the species' resources and consumers, respectively. That is, `resources` contains $\beta_{i1}\eta_{i1}, \beta_{i2}\eta_{i2}, ...\beta_{in}\eta_{in}$, and `consumers` contains $\eta_{1i}, \eta_{2i}, ...\eta_{ni}$. All η_{ij} and η_{ji} representing interactions that are not present in the food web are set to zero. `isProducer` is a Boolean variable that is true if the given species is a primary producer and false otherwise. Lastly, the class `Species` contains the function `computeDerivative()` that computes the derivative of the given species according to Eq. (2).

The class `Producer` contains the additional global variable `nProducer` which is the total number of primary producers in the food web. Furthermore, `growth` represents k_i and `computeDerivative()` computes the derivative according to Eq. (1).

The class `FoodWeb` is shown in Fig. S6b which is mainly created to keep track of indices during the integration. `feasible` and `stable` are true if the food web during a given invasion is feasible or linearly stable, respectively, and false otherwise. `prevIteration` is an integer that describes how the food web behaved after the previous invasion, and is used to optimise detection of non-convergent food webs. If the food web converged to the steady stated after the previous invasion, `prevIteration` is set to one. If the food web did not reach the steady states within the time limit, `prevIteration` is set to two (linearly stable food webs) or nine (unstable food webs). Finally, `prevExtinction` represents the previous species to go extinct.

S8 Varying the sampling distributions of α and η

In the main text we draw decay and interaction rates from uniform distributions (*see* Tab. 1). Here, we run the simulation for omnivorous food webs, drawing α and η from Gaussian and exponential distributions, respectively. The sample distributions are illustrated in Fig. S7. To avoid unphysical parameter values, a lower bound of 0.05 and 0.01 is imposed on the distributions of α and η, respectively. The η-distributions also have an upper bound of 1, whereas only the exponential α-distribution has an upper bound of 0.5. These bounds are the same as in the uniform case in Tab. 1. Fig. S8 show the resulting distributions of eigenvalues along the real axis for the three different sampling distributions of α and η. The eigenvalue distributions are practically identical, thus confirming that our results are robust to small changes in the distributions of parameters.

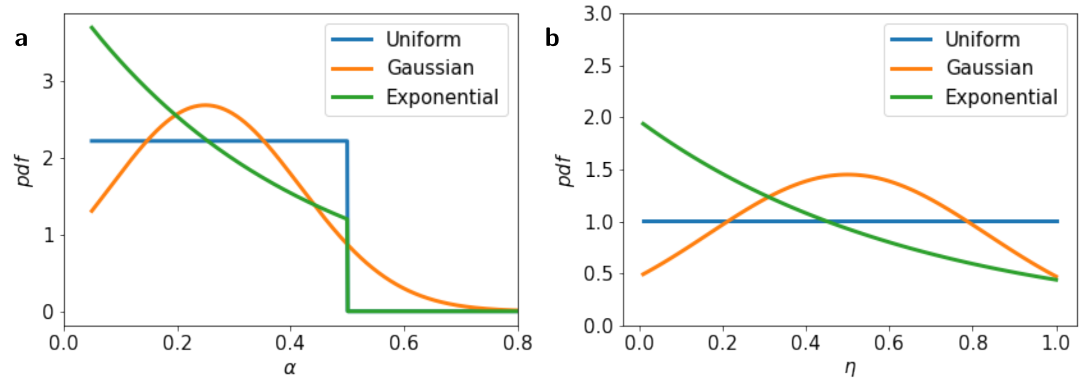

Figure S7: **Sampling distributions of α and η.** **a**, Sampling distributions of α. Gaussian distribution with $\mu = 0.25$ and $\sigma = 1/6$, exponential distribution with $\lambda = 2.5$. **b**, Sampling distributions of η. Gaussian distribution with $\mu = 0.5$ and $\sigma = 1/3$, exponential distribution with $\lambda = 1.5$.

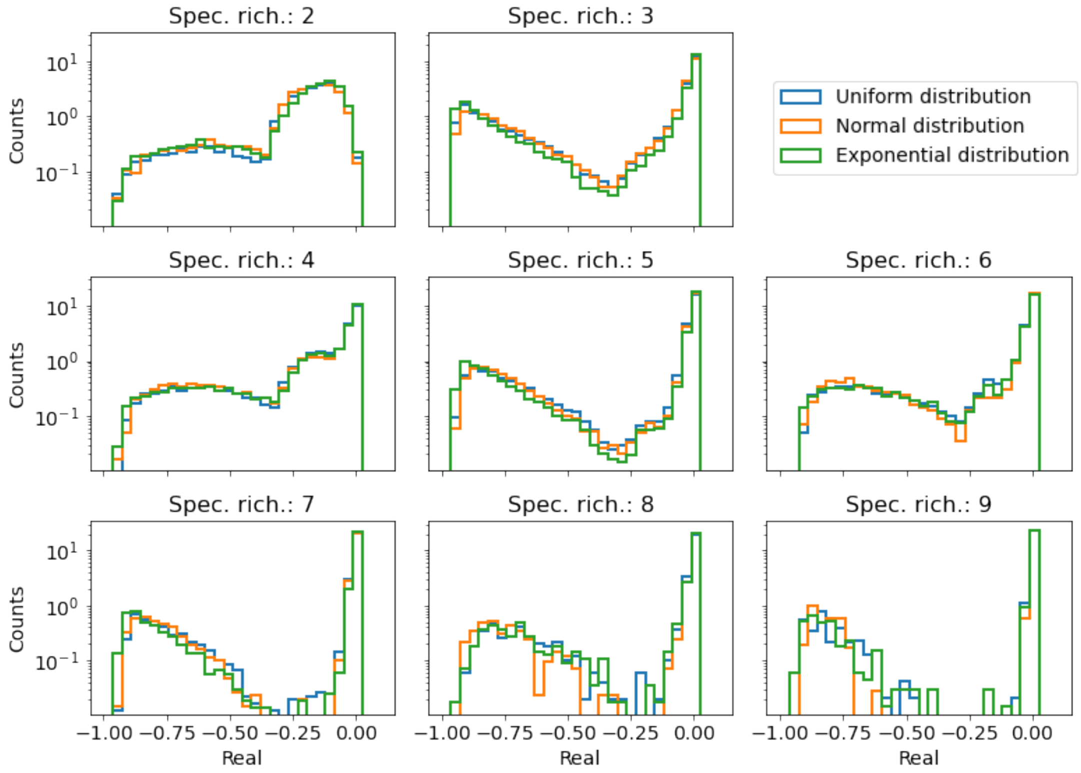

Figure S8: **Histograms of eigenvalues along the real axis for different sampling distributions of η and α.** The histograms are normalised frequency histograms of eigenvalues along the real axis for omnivorous food webs with species richness 2–9.

S9 Holling type-II response

The simulation is extended to allow consumption rates following Holling type-II functional response [3]

$$r(\eta_{ki}, S_i) = \frac{\eta_{ki} S_i}{1 + h\eta_{ki} S_i}, \tag{S14}$$

where S_i is the density of the resource species and η_{ki} is the link specific interaction strength between the resource i and consumer k as defined in Results and Discussion. The parameter h controls the significance of the type-II response. When $h = 0$ Eq. (S14) reduces to type-I functional response as used in Eqs. (1)–(2).

With consumption rates following Holling type-II functional response, the system of equations analogous to Eqs. (1)–(2) is no longer linear in S_i. Eq. (5) is therefore not applicable and neither are the convergence criteria described in Materials and Methods. A simpler algorithm is therefore employed in order to compute the eigenvalue spectra here. The food web is now considered to be converged if

$$\frac{|\dot{S}_i|}{S_i} \leq 10^{-10} \quad \forall i, \tag{S15}$$

and the densities that satisfy Eq. (S15) are plugged in as $\mathbf{S}(t)$ in Eq. (6). The overall procedure is shown in Algorithm 2.

Algorithm 2: Pseudo-code of the evolutionary algorithm with type-II functional response

```
initialize food web;
for each invasion do
    add new species;
    while not converged do
        integrate food web;
        update time;
        if extinction event then
            remove species;
            restart time;
        end
        if time ≥ 10^5 then
            break;
        end
    end
    compute eigenvalues;
end
```

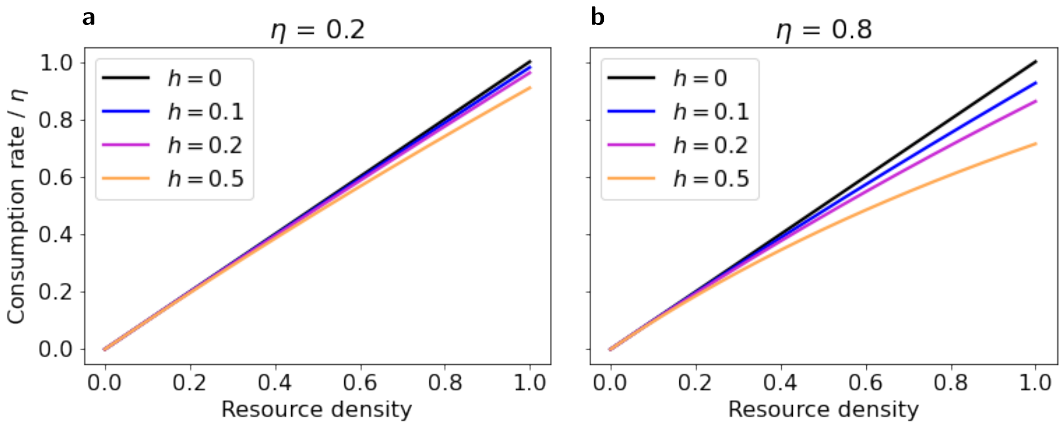

Figure S9: **Consumption rates for different values of h for $\eta = 0.2$ and $\eta = 0.8$. a**, $\eta = 0.2$ and the consumption rate changes little with h. **b**, $\eta = 0.8$ and the non-linearity of the consumption rate is more pronounced.

Since the type-II response community matrix eigenvalues are computed only from the food webs that satisfy Eq. (S15), we only obtain the stable eigenvalues. Starting from $h = 0$, we run the simulation for $h = 0.1, 0.2$ and 0.5. The effect on the consumption rate can be seen in Fig. S9 for two values of η. In interactions of low interaction strength

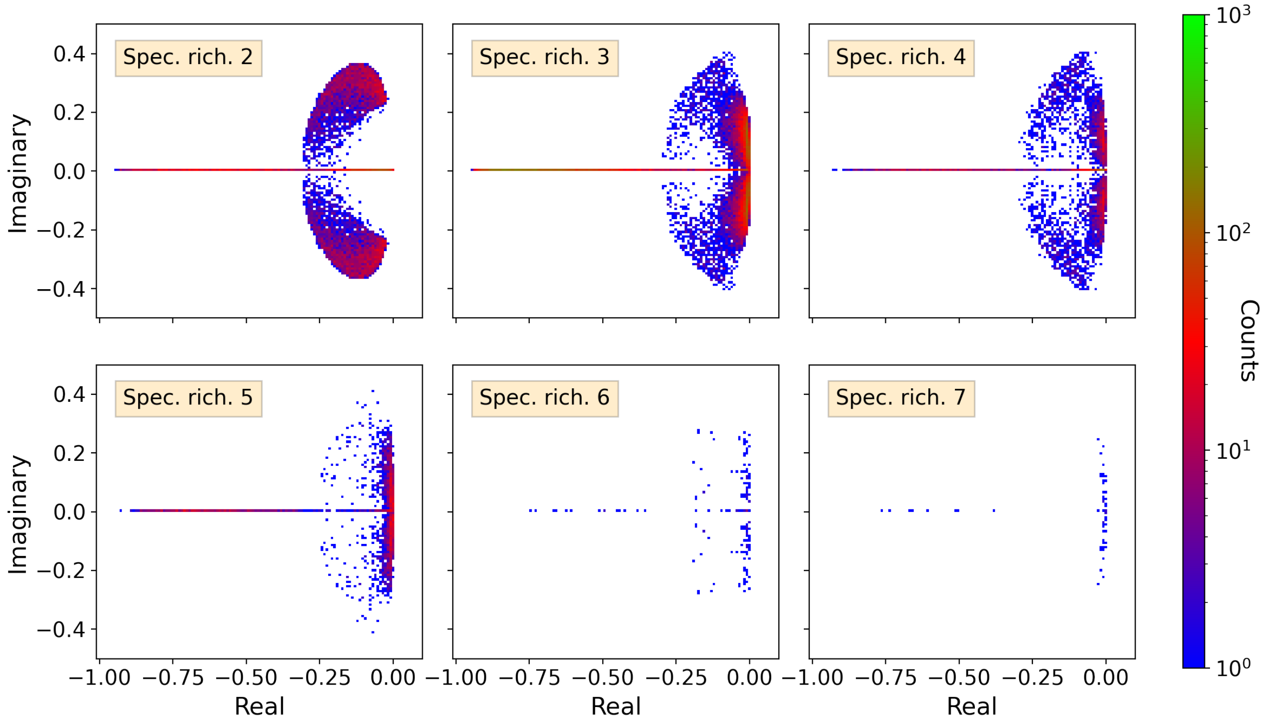

Figure S10: **Complex eigenvalues spectra of evolved omnivorous type-II response food webs with $h = 0.5$.**. Each panel represents the two-dimensional histogram in the complex plane. Species richness as labelled in panels and $\beta = 0.75$.

the non-linear effects are negligible for most h, whereas for high interaction strengths the non-linearity is pronounced for all h. As we increase h the average species richness decreases, and for $h = 0.5$ there are no convergent food webs of species richness higher than 7, though there are non-convergent food webs of up to 9 species. Fig. S10 shows the stable part of eigenspectra of food webs with species richness 2–7. The type-II spectra resemble the type-I spectra from Fig. S3, but are more skewed towards positive real values. It seems intuitive that interactions following type-II response would act as a dampening, thereby stabilising the food webs and allowing for food webs of higher species richness. To get some insight in why this is seemingly not the case, we study the spectrum of a food web with two species, analogous to Sec. S3. The steady states of this food web are

$$S_1^* = \frac{\alpha_2}{\eta\beta'}, \qquad S_2^* = \left[\frac{k}{\eta}\left(1 - \frac{\alpha_2}{\eta\beta'}\right) - \alpha_1\right]\frac{\beta}{\beta'}, \qquad \text{with } \beta' \equiv \beta - h\alpha_2, \tag{S16}$$

i.e. similar to the steady states of type-I response, with β' replacing β. From these we see that feasibility now requires

$$\textbf{1.}\quad \beta > h\alpha_2, \tag{S17}$$

$$\textbf{2}\quad \eta > \frac{1}{\beta'}\frac{\alpha_2}{1 - \alpha_1/k}. \tag{S18}$$

Since $\beta' \leq \beta$ for all h and α_2, we see that it requires a higher η to satisfy the feasibility criterion of Eq. (S18), compared that of type-I response.

Then we study the stability of this food web. First we compute the community matrix according to Eq. (6)

$$C_{11} = \left(k\left(1 - \frac{\alpha_2}{\beta'\eta}\right) - \alpha_1\right)\left(1 - \frac{\beta'}{\beta}\right) - \frac{k\alpha_2}{\beta'\eta}, \tag{S19}$$

$$C_{12} = -\frac{\alpha_2}{\beta} < 0, \tag{S20}$$

$$C_{21} = k\beta'\left(1 - \frac{\alpha_2}{\eta\beta'}\right) - \alpha_1\beta' > 0 \quad \text{(from feasibility)}, \tag{S21}$$

$$C_{22} = 0. \tag{S22}$$

Since $C_{22} = 0$ and $C_{12}C_{21} < 0$, the quadratic formula for the eigenvalues reduces to

$$2\lambda_\pm = C_{11} \pm \sqrt{C_{11}^2 - 4|C_{12}C_{21}|}, \tag{S23}$$

and we see that both eigenvalues are always stable when $C_{11} < 0$, because the square root is always smaller or equal to C_{11}. After some algebra we find the following criterion for stability

$$\eta < \frac{k/h}{k-\alpha_1}\frac{\beta + h\alpha_2}{\beta - h\alpha_2}. \tag{S24}$$

The RHS is always larger than 1 (for $h \leq 1$), which is the upper limit on η. Food webs of species richness 2 are therefore also always stable when we introduce Holling type-II response, given that $h \leq 1$. However, they might be unstable for $h > 1$.

We only use $h < 1$ in our simulations, and Eq. (S24) is always satisfied with our choice of parameters. Yet the upper limit on η decreases with h, meaning the system somehow becomes "less stable" with h. It therefore seems reasonable that larger food webs with type-II response can indeed be less stable than their type-I counterparts.

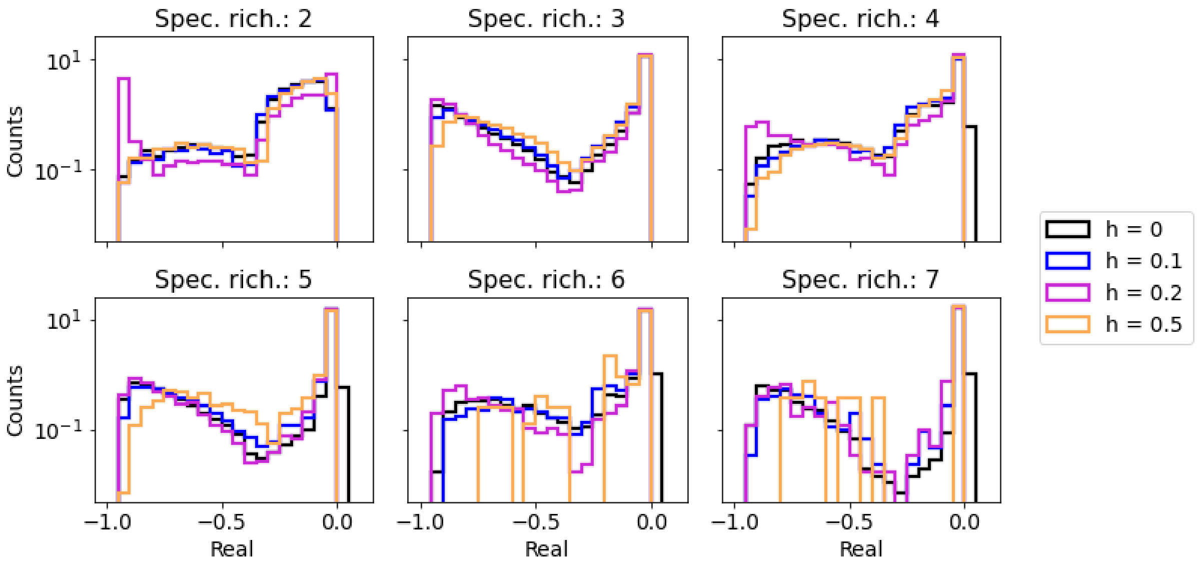

Figure S11: **Distribution of eigenvalues along the real axis**

Lastly, we plot the distributions of type-II eigenvalues along the real axis in Fig. S11. Despite a low number of eigenvalues from the simulation with $h = 0.5$, all distributions appear to follow approximately the same bi-modal form as in the case of $h = 0$. An exception is the pronounced peak near the lower limit for species richness 2 and $h = 0.2$. The origin of this peak is not investigated further.

S10 Matrix elements of the community matrix

From Eq. (6) we have the following mathematical expression for the community matrix

$$C_{ij} \equiv \frac{\partial}{\partial S_j} \dot{S}_i \left(\mathbf{S}(t)\right)\Big|_{\mathbf{S}=\mathbf{S}^*}.$$

We now compute the unique matrix elements explicitly, starting with the Lotka-Volterra equation for a primary producer (Eq. (1))

$$C_{ii} = k_i \left(1 - \sum_{j=1}^{n_1} S_j^* - S_i^*\right) - \alpha_i - \sum_{k=n_1+1}^{n} \eta_{ki} S_k^* \qquad \text{(Eq. (1) w.r.t. } S_i\text{)}, \tag{S25}$$

$$C_{ij} = -k_i S_i^* \qquad \text{(Eq. (1) w.r.t. } S_j,\ j \neq i\text{)}, \tag{S26}$$

$$C_{ik} = -\eta_{ki} S_i^* \qquad \text{(Eq. (1) w.r.t. } S_k\text{)}. \tag{S27}$$

Here, like in the main text, S_i and S_j, $i, j \in \{1, \ldots, n_1\}$ represent primary producers, and S_k, $k \in \{n_1 + 1, \ldots, n\}$ represents a consumer species. Then we compute the matrix elements corresponding to the Lotka-Volterra equation for all other species, S_k (Eq. (2))

$$C_{kk} = \sum_{m=1}^{n} \beta_{km} \eta_{km} S_m^* - \alpha_k - \sum_{p=n_1+1}^{n} \eta_{pk} S_p^* = 0 \qquad \text{(Eq. (2) w.r.t. } S_k\text{)}, \tag{S28}$$

$$C_{km} = \beta_{km} \eta_{km} S_k^* \qquad \text{(Eq. (2) w.r.t. } S_m,\ m \neq k\text{)}, \tag{S29}$$

$$C_{kp} = -\eta_{pk} S_k^* \qquad \text{(Eq. (2) w.r.t. } S_p,\ p \neq k\text{)}. \tag{S30}$$

Here S_m, $m \in \{1, \ldots, n\}$ represents a resource species of S_k and S_p, $p \in \{n_1 + 1, \ldots, n\}$ represents a consumer species of S_k. Since Eq. (2) is linear in S_k, we have that $C_{kk} = 0$ for all $k \in \{n_1 + 1, \ldots, n\}$. All matrix elements representing interactions that are absent in the present food web yield 0.

References

[1] Robert M May. Will a large complex system be stable? *Nature*, 238(5364):413, 1972.

[2] Andreas Knoblauch. Closed-form expressions for the moments of the binomial probability distribution. *SIAM Journal on Applied Mathematics*, 69(1):197–204, 2008.

[3] Crawford S Holling. Some characteristics of simple types of predation and parasitism1. *The Canadian Entomologist*, 91(7):385–398, 1959.
